# Calcifying nested stromal epithelial tumor of the liver in a patient with Klinefelter syndrome: a case report and review of the literature

**DOI:** 10.1186/s12957-018-1528-x

**Published:** 2018-11-19

**Authors:** Satoru Tsuruta, Norihisa Kimura, Keinosuke Ishido, Daisuke Kudo, Kentaro Sato, Tetsu Endo, Tadashi Yoshizawa, Aoi Sukeda, Nobuyoshi Hiraoka, Hiroshi Kijima, Kenichi Hakamada

**Affiliations:** 1 Department of Gastroenterological Surgery, Hirosaki University Graduate School of Medicine, 5, Zaifu, Hirosaki, Aomori 036-8562 Japan; 2 Department of Gastroenterology and Hematology, Hirosaki University Graduate School of Medicine, 5, Zaifu, Hirosaki, Aomori 036-8562 Japan; 3 Department of Pathology and Bioscience, Hirosaki University Graduate School of Medicine, 5, Zaifu, Hirosaki, Aomori 036-8562 Japan; 4 Division of Pathology and Clinical Laboratories, National Cancer Center Hospital, 5-1-1 Tsukiji, Chuo-ku, Tokyo, 104-0045 Japan

**Keywords:** Calcifying nested stromal epithelial tumor (CNSET), Hormone imbalance, Klinefelter syndrome, Liver, Neoplasm

## Abstract

**Background:**

Calcifying nested stromal epithelial tumor (CNSET) is a primary neoplasm of the liver, characterized by well-demarcated nests consisting of spindle and epithelioid cells with calcification and bone formation. An association of Cushing syndrome with CNSET has drawn attention, but the origin of CNSET has not been clarified.

**Case presentation:**

We report here the case of a 20-year-old male with Klinefelter syndrome who underwent liver resection for an increasing liver tumor that was pathologically diagnosed with CNSET. He was postoperatively followed up and received several examinations, and recurrences and extrahepatic lymph node metastases were detected on the 64th day after surgery. Chemoembolization and chemotherapy were not effective, leading to tumor progression with development of progressive liver failure, and the patient finally died 164 days after hepatectomy.

**Conclusions:**

This case suggests that an imbalance of hormones affects the genesis and progression of CNSET, and indicates the importance of closely following patients with CNSET by imaging with attention to hepatic recurrence and extrahepatic metastases.

## Background

Calcifying nested stromal-epithelial tumor (CNSET) is an uncommon primary hepatic tumor that is characterized by a nested morphologic growth pattern composed of spindled and epithelioid cells with various shape of calcification or ossification. Most liver cancer is hepatocellular carcinoma (HCC), followed by intrahepatic bile duct cancer. In 2001, Ishak et al. first described a non-hepatic and non-biliary tumor resembling CNSET [1]. This tumor is known by several other names, including ossifying stromal-epithelial tumor, desmoplastic nested spindle cell tumor of the liver (DNSTL), nested stromal epithelial tumor (NSET), and ossifying malignant mixed epithelial and stromal tumor [2–23]. As far as we are aware, 38 cases have been reported in the literature. These tumors have similar morphology, immunohistochemistry, and molecular profiles, and Misra et al. suggested that they may be related, but with a spectrum of morphologic features [24]. The reported tumors have been found predominantly in females and commonly in children, and most arose from the right hepatic lobe. In a number of cases, an association between these tumors and Cushing syndrome has also been described. Here, we report a case of postoperatively recurrent CNSET with aggressive clinical behavior and extrahepatic lymph node metastasis in a patient with Klinefelter syndrome. To our knowledge, this is the first case of a patient with CNSET concurrent with Klinefelter syndrome. CNSET is generally described as a tumor with low malignant potential, but the severe and progressive clinical course in our case indicates that the pathogenesis of CNSET may be related to hormone imbalance.

## Case presentation

The patient was a 20-year-old male who had been a low-birth-weight infant, and had a history of Klinefelter syndrome and pulmonary valve stenosis. He was introduced to our hospital for further examination of a liver tumor that was increasing in size. The tumor had been found incidentally after laboratory findings in a health checkup showed impairment of liver function. The patient had declined treatment due to his employment situation, and had instead been followed up for 1 year.

At the first visit, he was completely asymptomatic with normal vital signs. A physical examination revealed a palpable right upper mass without tenderness. No symptom related to Cushing syndrome was observed. In blood tests, hepatitis B virus surface antigen and hepatitis C virus antibody were negative. Liver function tests indicated mild dysfunction. Regarding tumor markers, serum alpha-fetoprotein (AFP) and carcinoembryonic antigen (CEA) were normal; however, neuron-specific enolase (NSE) was elevated.

**Fig. 1:**
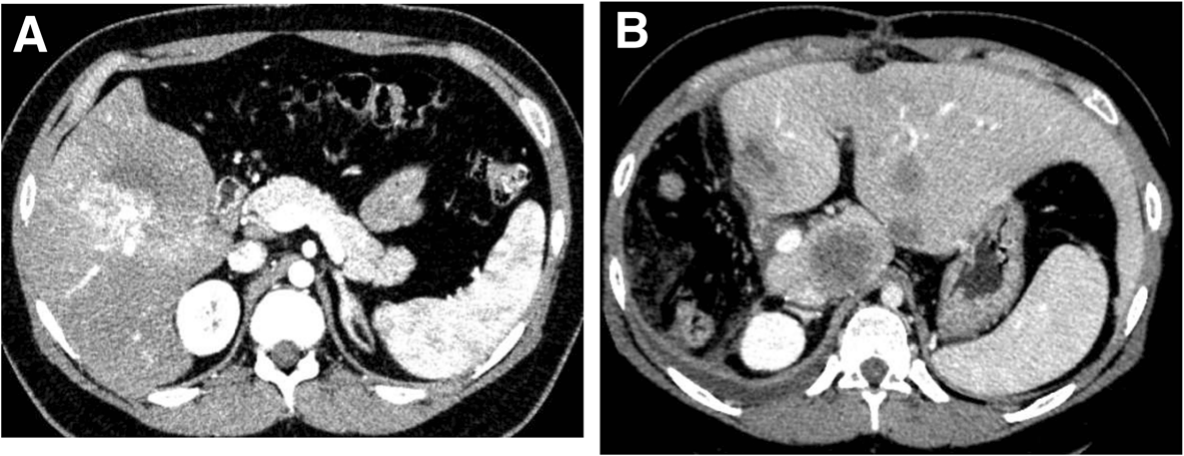
CT findings. **a** A large, heterogeneously enhanced mass with focal calcification was present in the right lobe of the liver. **b** Multiple recurrent lesions in the remnant liver and an enlarged para-aortic lymph node on postoperative day 62

Ultrasonography showed a large low-echoic solid tumor with a vertical diameter of > 80 mm with partial calcification implied by an acoustic shadow in an anterior lesion of the liver. A computed tomography (CT) scan of the chest, abdomen, and pelvis revealed an 81 × 76 × 72 mm large, heterogeneously enhanced mass in the right lobe of the liver with dense partial calcification (Fig. 1a). Subsequent positron emission tomography (PET)/CT showed a large hepatic mass in the right lobe with a maximum standardized uptake value (SUV) of 22.4 and no extrahepatic metastasis. In magnetic resonance imaging (MRI), most of the tumor was weakly enhanced in T1-weighted images and strongly enhanced in T2-weighted images. Part of the tumor had early enhancement and washout in enhanced MRI. These findings suggested HCC, and especially fibrolamellar HCC, but without evidence of distant metastasis.

Right hepatic lobectomy and cholecystectomy were performed 11 months after the initial detection of the tumor. The patient received no adjuvant chemotherapy or radiotherapy. The postoperative course was characterized by respiratory failure that required reintubation on postoperative day (POD) 2. X-ray and bronchofiberscopy showed pneumoniae due to pulmonary atelectasis and pulmonary edema. The subsequent hospital course was uneventful. On POD 7, a CT scan of the abdomen was interpreted as negative for hemoperitoneum and tumor recurrence, and the patient was discharged on POD 12.

The patient was followed up as an outpatient and received several examinations. On POD 62, a CT scan showed multiple, obscure, and circumscribed recurrent lesions in the remnant liver with contrast enhancement. The largest of these lesions had a diameter of 42 mm in segment 1 (S1) (Fig. 1b). In addition, a hypermetabolic para-aortic lymph node with possible metastasis was identified. On PODs 70 and 73, the patient underwent transcatheter arterial chemoembolization (TACE), but a second CT scan in the outpatient department on POD 84 revealed enlargement of recurrent tumors and the para-aortic lymph node. Chemotherapy (protocol for HCC) was started, but was unsuccessful because of side effects. At this time, there were no further surgical options and no other chemotherapy that was likely to be effective. Therefore, the patient received palliative care. The patient died 164 days after hepatectomy from tumor progression with development of progressive liver failure.

**Fig. 2:**
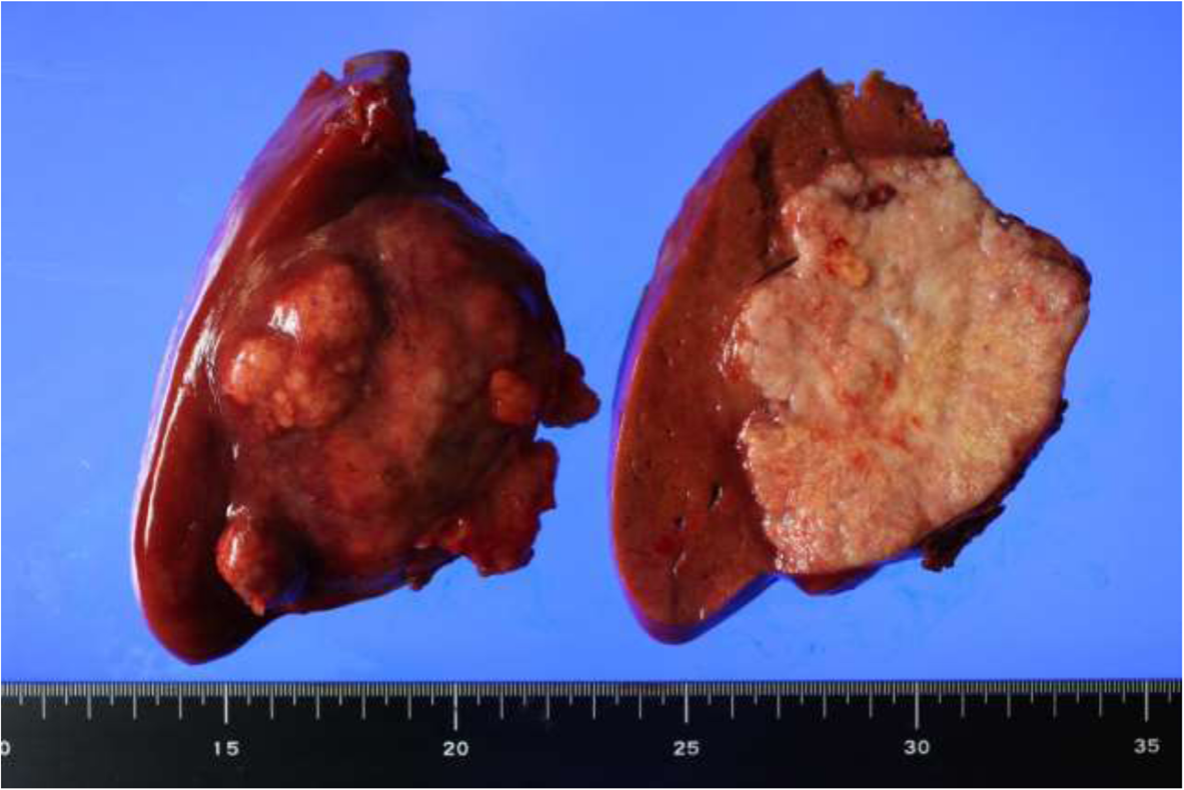
Gross findings for the lesion. An inspection showed a well-circumscribed solitary mass with multiple small calcifications that were sharply demarcated from surrounding liver parenchyma

**Fig. 3:**
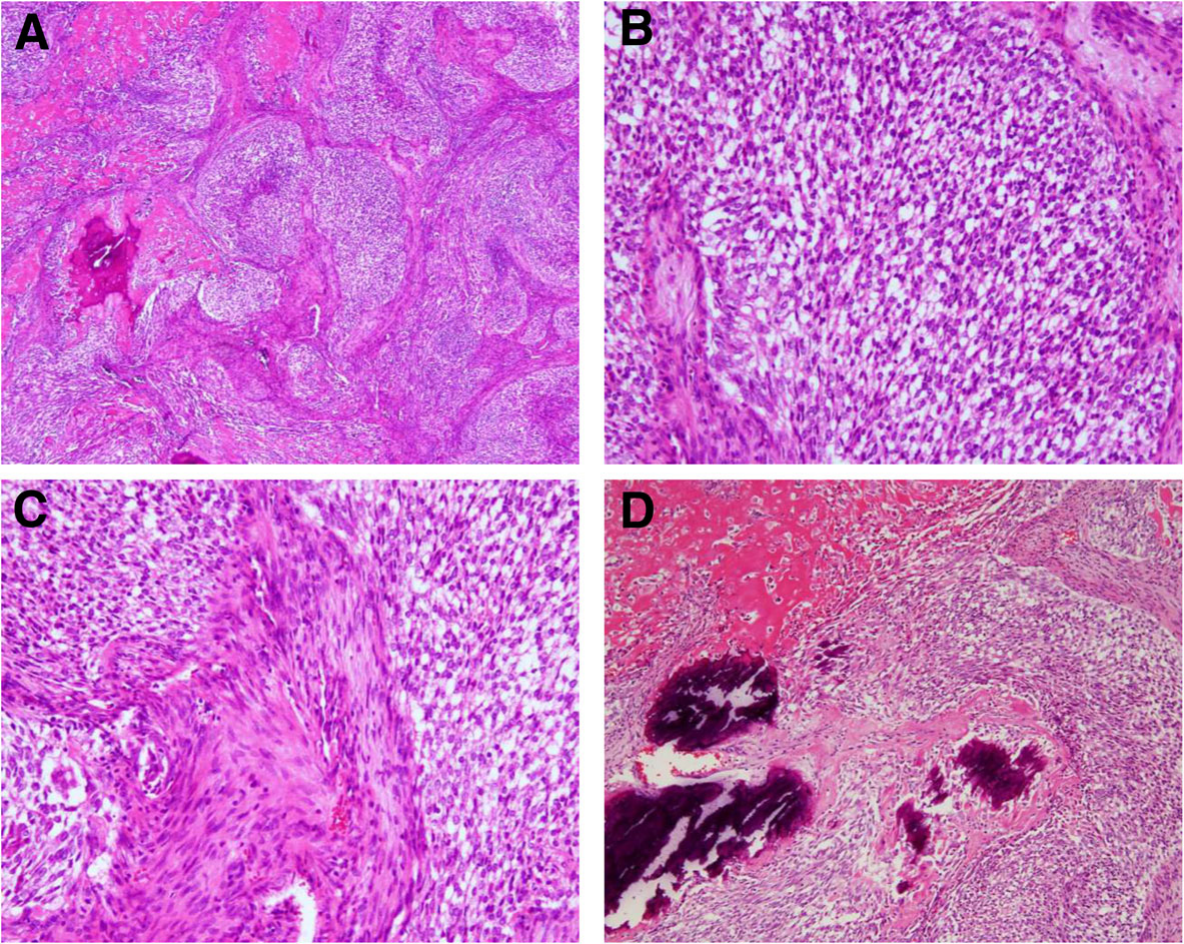
Histopathological findings for the tumor. **a** Nests of epithelial cells surrounded by a desmoplastic stroma with focal calcifications (× 40). **b** Epithelioid nests (× 200). **c** Desmoplastic stroma of spindle cells (× 200). **d** Necrosis and regions of calcification (× 100)

Grossly, the tumor was confined to the right liver lobe. The resected specimen weighed 1180 g. The lesion had a maximum diameter of 100 mm, and was a well-circumscribed solitary mass with multiple small calcifications that were sharply demarcated from surrounding uninvolved liver parenchyma (Fig. 2). The surgical margin was tumor-free. Microscopically, the tumor was characterized by an organoid arrangement of cellular nests of epithelioid cells and areas of sheet-like cell overgrowth (Fig. 3a). These cells had oval-like nuclei with no clear nucleolus and eosinophilic cytoplasm. Transition zones between epithelioid and spindle cells were observed, and a framework of spindle cells surrounded nests of epithelioid cells (Fig. 3b, c). Bile ducts were not intermingled with the tumor region. There were extensive regions of necrosis and calcification (or ossification) in the center of the tumor (Fig. 3d).

**Fig. 4:**
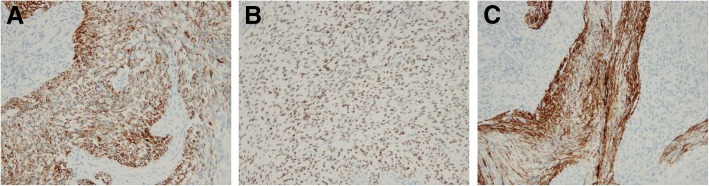
Immunohistochemical findings of the tumor. **a** Positive immunostaining with antibodies against cytokeratin AE1/AE3 in epithelioid cells (× 200). **b** Nuclear staining positive for WT-1 (× 200). **c** Positive immunostaining with α-smooth muscle actin (α-SMA) in stroma (× 200)

In immunohistochemical staining, epithelioid cells were positive for CD56, cytokeratin AE1/AE3 (focal), WT-1 (diffuse or dot-like in cytoplasm), β-catenin (diffuse in nucleus), vimentin, NCAM, and NSE (Fig. 4a, b). Spindle cells in mesenchymal components such as the septum were diffusely stained with α-smooth muscle actin (α-SMA) (Fig. 4c). The AFP level was within the normal range. Staining for glypican-3 was negative. The proliferation index on MIB-1 (Ki-67) immunostaining was < 5%. Staining was negative for hepatocyte paraffin-1, CK7, adrenocorticotropic hormone (ACTH), estrogen receptor (ER), and progesterone receptor (PR). The morphological and immunohistochemical features led to diagnosis of CNSET.

## Discussion and conclusions

CNSET of the liver is an uncommon tumor that was first described by Ishak et al. in 2001. Predicting the clinical behavior of this tumor is difficult because only a few cases have been reported under a variety of names (Table 1). The name “calcifying nested stromal-epithelial tumor” was proposed by Makhlouf et al. to incorporate all of the observed features of these tumors [7]. Our case provides support for this name.

**Table 1:** Reported cases of CNSET

No	Authors	Age	Sex	Symptoms	Surgical treatment	Chemotherapy	Outcome/follow-up	Associated history or disease
1	Heywood et al. [2] 2002	28	F	Incidental	Trisegmentectomy IVB, V, and VI wedge resection VII	☓	Recurrence/72 months	Persistent fetal hemoglobin, OCP
2	Hill et al. [4] 2005	2	M	Abdominal mass	Partial hepatectomy	Post	Alive/84 months	
3		6	F	Incidental	Gross excision	☓	Alive/58 months	
4		6	F	Incidental	Gross excision	☓	Alive/8 months	
5		14	F	Abdominal mass	Left lobectomy	Post	Recurrence/11 years	
6	Heerema-McKenney et al. [3] 2005	2	M	Incidental	Gross excision	☓	Alive/8 months	Nephroblastomatosis and Wilms tumor
7		3	F	Incidental	Gross excision	Pre/post	Alive/6 months	
8		4	M	Incidental	Enucleation	Post	Alive/36 months	
9		11	F	Cushingoid features, abdominal mass	Left lobectomy	☓	Alive/24 months	Cushing syndrome
10		12	F	Cushingoid feature, abdominal mass	Right hepatectomy	☓	Alive/168 months	Cushing syndrome
11		14	F	Ileus	Gross excision	Post	Recurrence/12 months	BWS, hypoplastic kidney, omphalocele
12	Brodsky et al. [5] 2008	17.5	F	Cushingoid feature, abdominal pain	Left lobectomy + partial hepatectomy	☓	Recurrence/12 months	Cushing syndrome
13	Wirojanan et al. [6] 2008	2	M	ND	Resection	Post	Alive/84 months	Fragile X syndrome
14	Meir et al. [8] 2009	2.5	F	Incidental	Right lobectomy	☓	Alive/8 months	Asymptomatic hydronephrosis
15	Makhlouf et al. [7] 2009	2	F	Incidental	Partial hepatectomy	☓	Alive/6 months	
16		14	F	Incidental	Partial hepatectomy	Post	Alive/264 months	
17		15	F	Incidental	Partial hepatectomy	☓	Alive/151 months	
18		16	M	Cushingoid feature	Partial hepatectomy	☓	Alive/56 months	Cushing syndrome
19		18	F	Incidental	Transplant	☓	Death/40 months (no recurrence)	
20		19	M	Incidental	No (needle biopsy)	☓	Lost to follow-up	
21		32	F	Incidental	Partial hepatectomy	☓	Alive/13 months	
22		33	F	Incidental	Partial hepatectomy	☓	Alive/14 months	
23	Rod et al. [9] 2009	17	F	Cushingoid feature, palpable right upper abdominal mass	Resection	☓	Alive/30 months	Cushing syndrome
24	Grazi et al. [10] 2010	25	F	Diarrhea and recurrent abdominal pain	Right lobectomy extending the caudate lobe segment IV	☓	Alive/6 months	OCP
25	Ramirez et al. [11] 2010	33	M	Unspecific abdominal pain and dyspepsia	Left lobectomy	☓	Alive/15 months	HBV(+)
26	Wang et al. [13] 2011	34	F	Incidental	Left lobectomy	☓	Alive/42 months	OCP
27	Hommann et al. [12] 2011	14	F	Incidental	Resection, transplantation	Post	Recurrence/28 months	Moderate hypoxic brain injury, omphalocele
28	Assmann et al. [14] 2012	16	M	Palpable abdominal mass	Transplantation	Pre	Alive/24 months	Cushing-like habitus
29		3	F	Unclear obstipation	Partial hepatectomy	Post	Alive/5 years	
30	Geramizadeh et al. [15] 2012	8	M	Cushingoid feature	Right extended hepatectomy	☓	Death 10 days (no recurrence)	Cushing syndrome
31	Ghodke et al. [16] 2012	9	M	Abdominal pain, fever, jaundice, weight loss, anorexia	Segmental hepatectomy	ND	Alive/12 months	
32	Malowany et al. [17] 2013	2	F	Incidental	Resection	ND	No recurrence	BWS
33	Procopio et al. [18] 2014	23	F	Abdominal distension and dyspepsia	Extended left hepatectomy	☓	Alive/21 months	OCP
34	Samarghandi et al. [19] 2015	11	F	Weight gain, increased appetite, abdominal pain	Unknown	ND	ND	
35	Schaffer et al. [20] 2016	14	F	Abdominal distention and swelling of cheeks	Transplant	☓	Alive/10 months	BWS, Cushing syndrome
36	Weeda et al. [21] 2016	16	M	Cushingoid feature, weight gain, distended abdomen	Trisegmentectomy	☓	Alive/13 years	Cushing syndrome
37	Khoshnam et al. [22] 2017	14	F	Cushingoid feature, abdominal swelling and pain	Transplantation	Pre	Alive/ND	BWS, Cushing syndrome
38	Tehseen et al. [23] 2017	13	F	Abdominal pain and distention, Cushingoid features	Transplantation	Pre	Alive/28 months	Developmental delay, Cushing syndrome
39	Our case 2017	20	M	Incidental	Right hepatectomy	Post	Death recurrence/2 months	Klinefelter syndrome

*ND* not determined, ☓ not administered, *OCP* oral contraceptive pill use, *BWS* Beckwith-Wiedmann syndrome

CNSETs occur predominantly in females (male:female ratio of 1:2.5) and pediatric patients (age range 2–34 years, mean 13.8 years), and most (22/31 lesions) arise from the right hepatic lobe. Most reported cases of CNSET were asymptomatic and the tumor was incidentally discovered in a physical examination or abdominal imaging, although in some cases, atypical abdominal symptoms were observed. Interestingly, seven of the patients presented with Cushing syndrome or Cushingoid symptoms because of ectopic ACTH production from the tumor, and Cushingoid features resolved after resection of the primary tumor. Cushing-like symptoms were not evident in our case. In addition, three cases were associated with Beckwith-Wiedmann syndrome [17, 20, 22]. To our knowledge, our case is the first report of CNSET with Klinefelter syndrome.

Klinefelter syndrome occurs in males with at least one Y chromosome and at least two X chromosomes. It is the most common sex chromosomal disorder in males and affects 1 in 660 men [25]. This syndrome was first reported by Klinefelter et al. in 1942 [26], and several additional conditions, characteristics, and abnormalities were subsequently described in a number of reports. Klinefelter syndrome is the most common major abnormality of sexual differentiation, with serum testosterone at a low to normal level and elevation of gonadotropins. The overall cancer risk does not differ from that in the general population, but cancers such as extragonadal germ cell tumors and breast cancer are seen more frequently in Klinefelter syndrome [27]. A pathogenetic link between Klinefelter syndrome and liver cancer has not been proposed, but Beures et al. reported a liver adenoma in a young patient with Klinefelter syndrome [28].

Four of the patients with CNSET (Table 1) had a history of use of oral contraceptives, which is of interest because high serum estradiol and low serum testosterone occur in Klinefelter syndrome and in oral contraceptive use. An association between hepatic adenoma and oral contraceptives has been proposed, based on suspected carcinogenic effects of estrogen and enzyme induction of progesterone [29]. Wang et al. also proposed a relationship between oral contraceptive use and development of CNSET in adult women [13]. There are no reports of a relationship of CNSET with sex hormones, but we suggest that CNSET may be related to imbalance of sex hormones. A relationship between oral contraceptive use and breast cancer risk has been shown, and Beaber found that long-term use of oral contraceptives may be more strongly related to a risk of ER− (× 3.5) and triple-negative (× 3.7) cancer compared to ER+ cancer, although the differences were not significant [30]. Our case was negative for ER in immunostaining. An imbalance of sex hormones, such as a high level of estradiol, might initiate occurrence and development of CNSET via a non-sex hormone receptor pathway, and it is possible that the constitutive imbalance of sex hormones affected the aggressive clinical behavior in our case.

In six of the cases of CNSET, patients had a history of hepatic calcification since childhood. Makahlouf et al. suggested that CNSET begins as a gradually enlarging small calcified lesion [7]. In our case, calcification of the liver was not observed in childhood. In blood tests, serum levels of AFP and CEA were in the normal range in all investigated cases. Imaging shows a typically large and well-circumscribed lesion with a macrolobulated margin, as in our case; with a distinctive large mass with heterogeneous enhancement and dense calcification on CT; and similar features with predominant T1 hypointensity and T2 hyperintensity on MRI. Dynamic postcontrast MRI may help to distinguish CNSET from other diseases with similar enhancement patterns. Radiologic differential diagnoses include hepatic vascular formation, fibrolamellar HCC, and hepatoblastoma [20]. Fibrolamellar HCC, which we first suspected in our case, is often detected at a similar age and has similar imaging findings with a central scar on CT and MRI. Calcifications are seen in 35–68% of cases of fibrolamellar HCC, but these tend to be small and fewer than three in number [31].

On gross examination, a CNSET is a lobulated mass with variable calcification and is generally well-circumscribed within the liver. Some previous reports presented no evidence of calcification. The size of the tumors has ranged from 2.8 to 30 cm, and have tended to be large. On the cut surface, the tumor might appear granular, homogeneous white or tan, with foci of softening, and cyst formation. Histologic analysis shows typical characteristics of well-demarcated nests of spindle and epithelioid cells surrounded by a desmoplastic stroma. Within the nests, tumor cells with epithelioid shapes have bland clear features. The desmoplastic stroma has morphologic characteristics of myofibroblasts, and the surrounding liver parenchyma largely shows no remarkable finding. Individual cell psammomatous calcification and regions of ossification have frequently been described in previous case reports.

Immunohistochemistry can also help with diagnosis of CNSET. The tumors tend to be positive for vimentin, pan-cytokeratin, and CD57. Staining for WT-1 protein in tumor cells is varied, with weak to moderate nuclear staining, dot-like paranuclear staining, and diffuse cytoplasmic staining. Nest cells are focally positive for NSE, CD56, and sometimes S-100. Stromal components of CNSET are consistently immunoreactive for α-SMA. The histological differential diagnoses of tumors with both epithelial and mesenchymal components and variable calcification include hepatoblastoma, synovial sarcoma, teratoma, desmoplastic round cell tumor (DSRCT), inflammatory myofibroblastic tumor of the liver, biliary rhabdomyosarcoma, metastatic Wilms tumor, and spindled carcinoid tumor [24].

Standard treatment for CNSET has not established, but all reported cases underwent gross total resection of the tumor, including wedge resection, partial hepatectomy, and hepatic lobectomy. In seven patients diagnosed with Cushing syndrome, cushingoid symptoms subsided after tumors producing ACTH were excised. Six cases with unresectable tumors received liver transplantation. A few cases received chemotherapy using a soft tissue sarcoma or hepatoblastoma protocol. However, the effect of using chemotherapy or radiotherapy has not been proved.

The prognosis of CNSET is unclear, but the tumor is normally slow-growing and of low malignant potential. In contrast to our patient, most cases have long-term survival after resection. Five cases had local recurrence after excision of the primary tumor and two had metastasis. Brodsky et al. described a case with extrahepatic lymph node metastasis after resection of the primary liver tumor [5]. Hommann et al. described an unresectable tumor in a 16-year-old girl who underwent hepatic transplantation, but had lung metastasis at 28 months postoperatively and died due to lung metastasis 37 months after transplantation [12]. Makhlouf et al. described a patient with two local recurrences that were successfully treated by radiofrequency ablation [7]. Our case had local recurrence in the liver and extrahepatic lymph node metastasis immediately after resection and showed more aggressive clinical behavior than most cases of CNSET. Therefore, this case suggests that patients with CNSET should be carefully followed by imaging study with close attention to hepatic recurrence and extrahepatic metastases.

There is no conclusive evidence for the origin of CNSET, but several hypotheses have been proposed. In addition to the potential link between CNSET and oral contraceptives, it has been hypothesized that CNSETs are derivatives of hepatic mesenchymal precursor cells with possible differentiation along a bile duct lineage, based on CD56-positive staining of bile ducts and tumor nests [20]. It was also noted that WT-1 expressed in CNSETs might affect transformation of mesenchymal to epithelial cells [3]. Based on our case, we suggest that a continual imbalance of hormones influences the pathogenesis of CNSET and leads to aggressive behavior after resection. However, there is also no evidence for the histogenesis of CNSET, and further studies of this tumor are needed.

In conclusion, we have presented a case of calcifying nested stromal epithelial tumor of the liver, an uncommon tumor characterized by well-demarcated nests of epithelial and spindle cells surrounded by myofibroblastic stroma and various calcifications. To our knowledge, this is the first reported case of CNSET with Klinefelter syndrome. Since this tumor presented with a very aggressive clinical course with recurrences and metastasis, the genesis and progression of CNSET may be related to hormone imbalance. Additionally, this case indicates the importance of careful follow-up with imaging and close attention to recurrence and metastases in a patient with CNSET. More studies are needed to improve the diagnosis and treatment of CNSET.

## Abbreviations

AFP: Alpha-fetoprotein
CEA: Carcinoembryonic antigen
CNSET: Calcifying nested stromal epithelial tumor
CT: Computed tomography
DNSTL: Desmoplastic nested spindle cell tumor of the liver
DSRCT: Desmoplastic round cell tumor
HCC: Hepatocellular carcinoma
MRI: Magnetic resonance imaging
NSE: Neuron-specific enolase
NSET: Nested stromal epithelial tumor
PET: Positron emission tomography
POD: Postoperative day
SUV: Standardized uptake value
TACE: Transcatheter arterial chemoembolization
α-SMA: α-Smooth muscle actin
